# Grossesse sur utérus cloisonné menée à terme: à propos d'un cas avec revue de la literature

**DOI:** 10.11604/pamj.2015.22.219.7790

**Published:** 2015-11-10

**Authors:** Osman Ali, Ihssane Hakimi, Adil Chanana, My Abdellah Baba Habib, Khalid Guelzim, Jaouad Kouach, Driss Moussaoui Rahali, Mohammed Dehayeni

**Affiliations:** 1 Service de Gynécologie-Obstétrique, Hôpital Militaire d'Instruction de Mohamed V, Rabat, Maroc

**Keywords:** Utérus cloisonné, utérus bicorne, utérus unicorne, échographie 3D, hystérosonographie 3D, septate uterus, Bicornuate uterus, horned uterus, 3D ultrasound, 3D hysterosonography

## Abstract

L'utérus cloisonné est la malformation utérine la plus fréquente, comptant pour 30 à 50% des cas, suivie par les malformations utérines de type utérus bicorne et utérus unicorne. Nous rapportons un cas d'utérus cloisonné total suspecté lors de l'examen obstétrical d'une parturiente en travail, et confirmé à l'exploration au cours d'une césarienne réalisé pour le même motif. L'intérêt de ce cas est de montrer le pronostic obstétrical chez les femmes fertiles porteuses de cette malformation utérine.

## Introduction

Les malformations utérines sont relativement fréquentes puisqu'elles concernent 3 à 4% de la population féminine [1–3]. La prévalence exacte puisque beaucoup de ces malformations sont asymptomatiques et que les techniques d′imagerie telles que l′échographie 3D, l′hystérosonographie 3D et l′IRM ne sont disponibles que depuis quelques années [2, 4]. Les malformations utérines semblent être diagnostiquées plus fréquemment dans certains groupes de patientes, par exemple lors d′un suivi pour infertilité ou pour fausses couches à répétition [2, 5]. Parmi elles, nous retrouvons les malformations utérines en particulier les utérus cloisonnés qui peuvent être découverts lors d′un examen d′imagerie ou au cours d′une intervention chirurgicale, ou encore, comme dans notre cas, suspecté lors d'un examen obstétrical et confirmé à l'exploration utérine lors d'une sa césarienne. Nous rapportons ici un cas d′utérus cloisonné total découvert lors d'une césarienne indiquée après découvert d'un septum vaginal épais et deux orifice cervicaux simultanément dilatées découvert chez une primigeste en travail.

## Patient et observation

**Figure 1:**
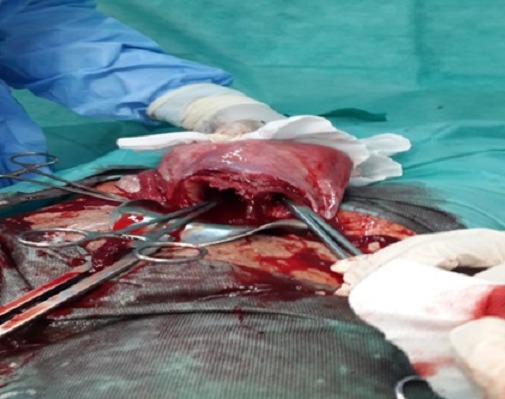
Une cloison séparant la cavité uterine

**Figure 2:**
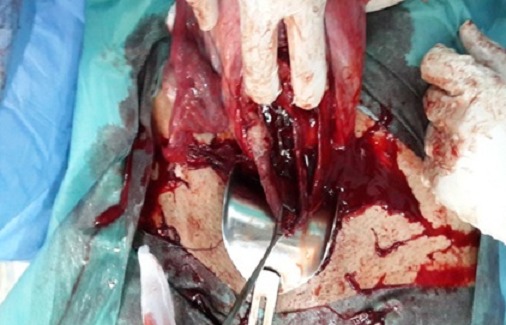
Cloison séparant le col utérin

**Figure 3:**
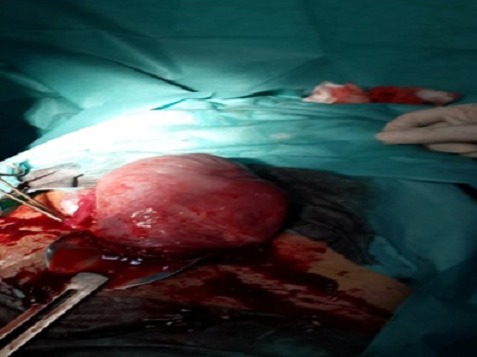
Le fond utérin ne presentant aucune anomalie

Il s'agit de Mme S H, primigeste de 21 ans de groupe O RH + sans antécédent particulier notamment, pas d'antécédent de pathologie urinaire, pas de dysménorrhée primaire ni de dyspareunie, ménarche à 13 ans; cycle régulier 5 jours sur 30, admise à 39SA et 3j pour prise en charge de son accouchement. L'examen général trouve une patiente en bon état général; normo tendue et apyrétique. L'examen obstétrical trouve une hauteur utérine à 33 cm des bruits cardiaques fœtaux positifs et réguliers. L'examen au speculum et le toucher vaginal trouve un septum vaginal épais avec deux orifices cervicaux dilatés chacun a 2 cm, effacés à 80pour cent avec une présentation céphalique fixée perçu à travers les deux orifices non perçu dans un orifice et dans l autre a travers la cloison, les membranes étaient intacte le bassin cliniquement normal. La césarienne a été indiquée permettant l'extraction d'un nouveau née de sexe masculin Apgar 10/10, pesant 3300 g. L'exploration per-opératoire après extraction à objectivé un utérus asymétrique avec 2 hémi-matrices celle contenant la grossesse plus volumineuse que l'autre, deux orifices cervicaux, et deux hémi-vagins séparés par un septum épais. a l'exploration il s'agit en réalité d'un un utérus cloisonné total (Figure 1, Figure 2, Figure 3) avec cloison épaisse qui s’étend de fond utérin jusqu’ au vagin, c'est un seul utérus il n y a pas les deux corne divergente avec la vessie qui s'insinue entre les deux et qui défini l utérus bicorne bi cervical. Aucun geste à visé thérapeutique portant sur la malformation n'a été réalisé. Le post parfum était sans particularité et un uro-scanner à été demandé pour le dépistage d’éventuelle malformation urinaire associée est revenu sans anomalies.

## Discussion

La prévalence des anomalies utérines congénitales dans la population est estimée entre 1 et 4% selon les études [2, 4]. Quoique cette prévalences reste inexact vu le caractères asymptomatique de ces malformations.le diagnostique est posé le plus fréquemment chez les patientes suivies pour infertilité, pour fausses couches à répétition ou pour accouchements prématurés, dans notre cas la patiente était asymptomatique dans sa vie et durant cette grossesse et le diagnostique a été posé à l'examen clinique faite à l'admission pour la prise en charge de son accouchement à terme. Il reste important d’évoquer le diagnostique chez l'adolescente qui consulte pour une aménorrhée primaire, une dysménorrhée ou dyspareunie. L'organogenèse des voies génito-urinaires permet d'interpréter et de classer les malformations génitales. Quatre phases peuvent être schématisées: la première phase urinaire (3^e^, 4^e^ et 5^e^ semaines) comporte la formation des canaux de Wolff et leur progression vers le cloaque, le développement des bourgeons urétéraux en direction des blastèmes rénaux; la deuxième phase, génitale et urinaire (6^e^, 7^e^, 8^e^ et 9^e^ semaines) comporte l'achèvement de l'appareil urinaire par l'ascension et la rotation des reins, à partir de la 9^e^ semaine l'organogénèse urinaire est donc achevée; la formation des canaux de Müller et leur progression vers le sinus génital peut débuter; la troisième phase, génitale, de l'accolement des deux canaux de Müller s’étale sur les 10^e^, 11^e^ et 12^e^semaines; cette phase est responsable de la morphologie externe des voies génitales; la quatrième et dernière phase est celle de la résorption de la cloison d'accolement des canaux de Müller (13^e^ à 17^e^ semaine). La résorption commence au niveau de l'isthme avant la fin de la phase d'accolement, s’étend rapidement vers le bas et lentement vers le haut. Cette phase de résorption est responsable de la morphologie interne des voies génitales. Le type des malformations est lié à la date de survenue de l'agent tératogène au cours de l'organogenèse: entre trois et six semaines, le canal de Müller n'existe pas encore. On observera une aplasie utérine, un utérus unicorne avec agénésie rénale unilatérale; à la sixième semaine, le canal de Müller se développe. Une anomalie apparaissant entre six et neuf semaines entraînera un utérus pseudo unicorne. Entre dix et treize semaines, les deux canaux de Müller se rapprochent de la ligne médiane. Les anomalies observées sont un défaut de fusion des deux canaux de Müller, à l'origine des utérus bicornes; après treize semaines, on observe un trouble de la résorption de la cloison à l'origine des utérus cloisonnés. La classification des malformations utérine La plus utilisée en France est la classification de Muset, établie en 1964 [4]. La classification internationale est celle de l'American Fertility Society (AFS) de 1988 [6]. Elle est la plus utilisée dans la littérature.

***Aplasies utérines (AFS classes I et II)*** Aplasies bilatérales complètes: type 0: organes urinaires et génitaux absents; type 1: deux ovaires isolés absence d'appareil génito-urinaire; type 2: type 1 avec en plus 2 ébauches de trompes; type 3: est constitué par les annexes; aplasies bilatérales incomplètes: syndrome de Rokitansky-Kuster-Hauser (classe Ic); aplasies unilatérales complètes: unicorne vrai (classe Iid); aplasies unilatérales incomplètes: pseudo unicorne (classes Iib et Iic).

***Hémi-utérus ou hémimatrices (AFS classes III et IV)*** Bicorne bicervical avec rétention menstruelle unilatérale; bicorne bicervical perméable; bicorne unicervical.

Utérus cloisonné (AFS classes V et VI) Cloisonné total; cloisonné subtotal; cloisonné corporel; cloisonné fundique; cloisonné cervical

***Utérus communicants (AFS classe II)*** Cloisonné total communicant; communicant bicorne bicervical avec rétention menstruelle unilatérale; cloisonné communicant corporel et bicervical.

En dehors de la grossesse, la malformation utérine peut être découverte dans le cadre d'un bilan d'aménorrhée primaire; de dysménorrhée primaire invalidante; de stérilité d'infécondité; d'accouchements prématurés à répétition, de dyspareunie. L'anomalie découverte au cours de l'examen clinique peut être: une absence de vagin; un bombement de la paroi latérale du vagin; une cloison vaginale; une bifidité cervicale ou même l'absence de col [6]. Pendant la grossesse, on peut être amené à diagnostiquer une malformation: lors d'une interruption prématurée de la grossesse; devant une présentation dystocique à répétition; lors d'un accident de la délivrance (hémorragie ou rétention); et plus rarement devant la survenue d'accidents: hémopéritoine par rupture d'une corne utérine rudimentaire gravide d'un utérus pseudo unicorne; ou syndrome abdominal aigu, par torsion d'un utérus gravide (absence ligamentaire congénitale). Dans notre cas, la patiente a été découverte à terme lors de l'examen faite à son admission pour la prise en charge de son accouchement.

Dans le bilan d'une malformation génitale Les différentes techniques utilisées sont l’échographie en 2D ou 3D, l'hystérosonographie, l'hystérosalpingographie, l'IRM, l'hystéroscopie et la laparoscopie [7]. Ces différentes techniques peuvent être combinées entre elles. L’échographie 3D et l'IRM sont actuellement les techniques montrant les meilleurs résultats en termes de sensibilité et spécificité [8, 9]. L’évaluation des malformations utérines doit être complétée par une imagerie rénale pour détecter les malformations des voies urinaires, fréquemment associées L’échographie doit toujours être réalisée, car c'est la seule exploration qui permet une évaluation précise endo- et exo-utérine. En pratique routinière, sa sensibilité reste peu importante (30 à 40%) et directement liée à l'expérience de l’échographiste. En revanche, orientée, notamment dans le cadre d'un bilan d'infertilité, elle doit permettre de définir l'existence ou non d'une cloison; dans ce cas, l'hystéroscopie doit être préférée à l'hystérographie. L’évaluation de la cloison utérine (hauteur, épaisseur, vascularisation) et des lésions associées est en effet plus pertinente en vision directe qu'au travers d'un écran radiographique. La cœlioscopie doit être exceptionnellement pratiquée et réservée aux seules observations où le diagnostic reste hésitant entre utérus cloisonné et utérus bicorne. Lorsque le diagnostic de malformation utérine est posé en début de grossesse, le traitement ne sera que préventif (repos, maturation pulmonaire, surveillance échographique de la croissance fœtale et de la compétence cervicale) [10]. Le cerclage cervical ne devrait être proposé qu′en cas d′incompétence cervicale prouvée, ce que l′on observe dans 25-30% des cas de malformations utérines [11–13].

L'indication d'une cure chirurgicale par voie endoscopique des cloisons utérines a obtenu actuellement un consensus général. Il s'agit en effet d'une intervention simple dont la morbidité n'est pas importante qui entraîne une hospitalisation réduite et dont les résultats sont comparables à ceux de l'hystéroplastie par voie abdominale. Les indications se sont de ce fait modifiées. Actuellement, l'indication opératoire par voie endoscopique semble être plus rapidement proposée (avortement tardif mais aussi précoce), parfois même en l'absence d'antécédent obstétrical.

Les équipes s'intéressant à la procréation médicalement assistée (PMA) proposent ainsi le traitement systématique de toutes les cloisons, même les plus discrètes (éperon) avant d'envisager la réalisation d'une PMA (fivette ou autre procédé). L'indication d'une hystéroplastie par voie endoscopique ne doit cependant pas être généralisée à tous les cas d'utérus cloisonnés pour les raisons suivantes: l'issue d'une grossesse ne peut jamais être prévue de façon formelle en présence d'un utérus cloisonné. Les observations de grossesse évoluant de façon normale jusqu'au terme, la naissance d'enfants de poids normal voire macrosomie malgré le handicap d'une cloison utérine, justifient une certaine prudence dans les indications systématiques de section endoscopique d'une cloison utérine; le pronostic ultérieur de fécondité est parfois grevé d'une morbidité iatrogène: destruction d'un ostium tubaire, fragilisation du fond utérin avec rupture utérine lors d'une grossesse ultérieure; l’évolution des grossesses est quasi normale en présence d'un utérus cloisonné total. Les indications d'hystéroplastie endoscopique recommandés sont guidées par les antécédents obstétricaux et l’état anatomique: antécédents d'avortement spontané tardif et semi-tardif (après la 12e semaine); antécédents d'avortement spontané du 1er trimestre après avoir éliminé une origine chromosomique ou hormonale; stérilité persistante en l'absence d'autres facteurs pouvant expliquer l'infertilité; présentation dystocique ayant entraînée une césarienne; cloison utérine partielle. Les hystéroplasties de principe ne sont pas recommandés en l'absence d'antécédents obstétricaux et d'infertilité et en présence d'une cloison utérine totale uni- ou bicervicale.

## Conclusion

Les malformations utérines congénitales sont relativement fréquentes et souvent asymptomatiques. Leur incidence exacte reste difficile à évaluer. Peuvent se manifester sous la forme de troubles gynécologiques ou avoir un impact sur la reproduction. Chaque clinicien doit rechercher une malformation utérovaginale en présence d'une aménorrhée primaire, de douleurs abdominales, de fausses couches à répétition et dans certaines issues obstétricales défavorables Il convient de rappeler que lors du diagnostic de malformation utérine, une imagerie des voies urinaires devrait être effectuée en raison des anomalies associées fréquentes.

## References

[1] Saravelos SH, Cocksedge KA, Li TC (2008). Prevalence and diagnosis of congenital uterine anomalies in women with reproductive failure: a critical appraisal. Hum Reprod Update..

[2] Raga F, Bauset C, Remohi J (1997). Reproductive impact of congenital Mullerian anomalies. Hum Reprod..

[3] Troiano RN, McCarthy SM (2004). Mullerian duct anomalies: imaging and clinical issues. Radiology..

[4] Nahum GG (1998). Uterine anomalies: How common are they, and what is their distribution among subtypes?. J Reprod Med..

[5] Poncelet C, Aissaoui F (2007). Malformations utérines et reproduction. Gynecol Obstet Fertil..

[6] The American Fertility Society classifications of adnexal adhesions (1988). Distal tubal occlusion, tubal occlusion secondary to tubal ligation, tubal pregnancies, mullerian anomalies and intrauterine adhesions. Fertil Steril.

[7] Deutch TD, Abuhamad AZ (2008). The role of 3-dimensional ultrasonography and magnetic resonance imaging in the diagnosis of mullerian duct anomalies: a review of the literature. J Ultrasound Med..

[8] Salim R, Woelfer B, Backos M, Regan L, Jurkovic D (2003). Reproductibility of three-dimensional ultrasound diagnosis of congenital uterine anomalies. Ultrasound Obstet Gynecol..

[9] Woelfer B, Salim R, Banerjee S (2001). Reproductive outcomes in women with congenital uterine anomalies detected by three-dimensional ultrasound screening. Obstet Gynecol..

[10] Airoldi J, Berghella V, Sehdev H, Ludmir J (2005). Transvaginal ultrasonography of the cervix to predict preterm birth in women with uterine anomalies. Obstet Gynecol..

[11] Acien P (1993). Reproductive performance of women with uterine malformations. Hum Reprod..

[12] Golan A, Langer R, Neuman M (1992). Obstetric out-come in women with congenital uterine malformations. J Reprod Med..

[13] Golan A, Langer R, Wexler S (1990). Cervical cerclageits role in the pregnant anomalous uterus. Int J Fertil..

